# Health Care Language Models and Their Fine-Tuning for Information Extraction: Scoping Review

**DOI:** 10.2196/60164

**Published:** 2024-10-21

**Authors:** Miguel Nunes, Joao Bone, Joao C Ferreira, Luis B Elvas

**Affiliations:** 1 ISTAR Instituto Universitário de Lisboa (ISCTE-IUL) Lisbon Portugal; 2 Select Data Anaheim, CA United States; 3 Department of Logistics, Molde, University College Molde Norway; 4 INOV Inesc Inovação Instituto de Novas Tecnologias Lisbon Portugal

**Keywords:** language model, information extraction, healthcare, PRISMA-ScR, scoping literature review, transformers, natural language processing, European Portuguese

## Abstract

**Background:**

In response to the intricate language, specialized terminology outside everyday life, and the frequent presence of abbreviations and acronyms inherent in health care text data, domain adaptation techniques have emerged as crucial to transformer-based models. This refinement in the knowledge of the language models (LMs) allows for a better understanding of the medical textual data, which results in an improvement in medical downstream tasks, such as information extraction (IE). We have identified a gap in the literature regarding health care LMs. Therefore, this study presents a scoping literature review investigating domain adaptation methods for transformers in health care, differentiating between English and non-English languages, focusing on Portuguese. Most specifically, we investigated the development of health care LMs, with the aim of comparing Portuguese with other more developed languages to guide the path of a non–English-language with fewer resources.

**Objective:**

This study aimed to research health care IE models, regardless of language, to understand the efficacy of transformers and what are the medical entities most commonly extracted.

**Methods:**

This scoping review was conducted using the PRISMA-ScR (Preferred Reporting Items for Systematic reviews and Meta-Analyses extension for Scoping Reviews) methodology on Scopus and Web of Science Core Collection databases. Only studies that mentioned the creation of health care LMs or health care IE models were included, while large language models (LLMs) were excluded. The latest were not included since we wanted to research LMs and not LLMs, which are architecturally different and have distinct purposes.

**Results:**

Our search query retrieved 137 studies, 60 of which met the inclusion criteria, and none of them were systematic literature reviews. English and Chinese are the languages with the most health care LMs developed. These languages already have disease-specific LMs, while others only have general–health care LMs. European Portuguese does not have any public health care LM and should take examples from other languages to develop, first, general-health care LMs and then, in an advanced phase, disease-specific LMs. Regarding IE models, transformers were the most commonly used method, and named entity recognition was the most popular topic, with only a few studies mentioning Assertion Status or addressing medical lexical problems. The most extracted entities were diagnosis, posology, and symptoms.

**Conclusions:**

The findings indicate that domain adaptation is beneficial, achieving better results in downstream tasks. Our analysis allowed us to understand that the use of transformers is more developed for the English and Chinese languages. European Portuguese lacks relevant studies and should draw examples from other non-English languages to develop these models and drive progress in AI. Health care professionals could benefit from highlighting medically relevant information and optimizing the reading of the textual data, or this information could be used to create patient medical timelines, allowing for profiling.

## Introduction

The health care sector generates a vast amount of structured and unstructured data, including images from medical exams, text written in electronic medical records (EMRs) or Electronic Health Records (EHRs), and structured data from relational databases that store patient and admission information, as well as all the data collected during a patient’s hospitalization [1]. Approximately 30% of the world’s data volume is generated by the health care sector, and projections indicate that by 2025, the compound annual growth rate of data for health care will reach 36% [2].

Medical texts present several challenges due to the use of unfamiliar context-specific terminologies that differ from everyday language. In addition, physicians often use abbreviations and acronyms to save time and space. However, the same abbreviation can have different meanings, adding an additional layer of complexity when trying to understand the content of medical texts [3]. All these characteristics pose challenges when attempting to apply artificial intelligence (AI) techniques to interpret the text.

In the field of natural language processing (NLP), the introduction of transformers [4] has revolutionized the field, achieving state-of-the-art performance for numerous NLP tasks [5]. Their general architecture comprises an encoder, which receives the input and builds a representation of it, and a decoder that uses the encoder’s representation along with other inputs to generate a target sequence. The introduction of the self-attention mechanism further revolutionized NLP by allowing the model to weigh the importance of different words in a sentence regardless of their position. This enables better handling of long-range dependencies compared with traditional deep learning (DL) architectures like recurrent neural networks (RNNs) and long short-term memory Networks [6]. In the context of medical text, transformers excel in interpreting and extracting medically relevant information by effectively handling context and meaning, even in complex and specialized language.

Transformers can be trained as language models (LMs) on raw text in a self-supervised manner, enabling them to develop a statistical understanding of the text they were trained on [7]. However, the benefits of this approach are only fully realized when fine-tuning a downstream task.

Another important concept is called domain adaptation, which stands for the process of adapting or adjusting something to be suitable within a different domain or context. In the field of machine learning (ML), domain adaptation is used to align the disparity between domains so that the trained model can generalize into the domain of interest [8]. For transformers, domain adaptation involves continuing the pretraining of an LM with text data from a different domain than the one it was originally trained on [9]. This approach allows for leveraging the learning capabilities of general-scope LMs and refining them for specific contexts. For example, if we consider a general-scope LM, one that was trained using textual data from various domains, and continue its pretraining with health care–specific textual data, it will help the LM to refine its understanding of the health care data, leading to improvements when fine-tuning the LM for downstream tasks related to health care. To explore this further, we can take a health care LM who was trained using EMRs from a hospital and continue its pretraining using only text from patients with a specific disease. It will allow the LM to adjust its weights and become more precise when interpreting texts related to that particular disease.

An example of domain adaptation is the BioBERT model [10], which resulted from the continuation of the pretraining of the Bidirectional Encoder Representations from Transformers (BERT) [10] model on biomedical text. The BioBERT model outperformed its predecessor in biomedical named entity recognition (NER), relation extraction, and question-answering tasks. Alzheimer’s Disease-BERT [11] and CancerBERT [12] are 2 examples of applying domain adaptation to a more restricted domain. Both models outperformed their respective baselines on downstream tasks related to their respective diseases. Summing up, performing domain adaptation for the health care sector appears inevitable to improve results, for example, for information extraction (IE) models, where a better understanding of medical terminologies and lexicon would make it easier to identify and extract information [13].

The European Portuguese (PT-PT) language does not generate the same amount of data as the English language, resulting in limitations in the literature and the published models. A study published in 2023 by the Ernst & Young Audit highlights the following areas where AI can play a relevant role in Portugal’s health care; disease diagnoses, precision medicine, remote monitoring and prevention, data management and hospital efficiency, and health policies [14]. Recently, a project was launched in Portugal, funded by the European Union, with the aim of creating PT-PT NLP solutions for the health care sector. Under this scope, the objective is to create PT-PT medical LM and IE models to automatically identify medically relevant entities.

Therefore, in this study, we aim to present a scoping literature review (SLR), in which we will begin by exploring the creation of health care LMs through domain adaptation and analyze their results. In addition, we aim to focus on the geographical domain to understand the current state-of-the-art for the Portuguese language and compare it to other, potentially more developed, languages to identify further steps. We also want to explore IE models in the health care sector, regardless of their data language, to understand the most commonly extracted medical entities and the methods used in doing so. Despite the literature being rich in studies focused on health care large language models (LLMs), there is a lack of studies that evaluate the current state-of-the-art of health care LMs not only in English but also in other less-resourced languages. This will enable us to grasp how the community is using the capabilities of transformers and whether the advantages of using them are indeed present in the health care domain. In addition, researchers will have 1 study about health care LMs that could guide their path and help them understand how the literature has developed in their respective languages. Finally, we will present the corresponding discussion and the conclusion drawn from the SLR.

## Methods

### Overview

To complete our goal, we have conducted an SLR to gain a better understanding of the research conducted in the application of health care–domain LMs and the development of IE models within the health care domain. In the first stage, our study encompasses health care–domain LMs in various languages, with a focus on the Portuguese language. In the second stage, we searched for studies related to IE models to evaluate the methods most frequently used. In terms of methodology, we followed the PRISMA-ScR (Preferred Reporting Items for Systematic reviews and Meta-Analyses extension for Scoping Reviews) [15] to ensure a systematic and transparent approach in conducting and reporting our scoping review.

### Search Strategy and Inclusion Criteria

This SLR was conducted in November 2023 and focused exclusively on studies and reviews published in journals within the last 5 years (2019-2023) that were written in English or Portuguese. It was not an arbitrary data range since limiting the search to the last 5 years ensures that the review includes the most recent and relevant studies, reflecting the latest advancements, technologies, and methodologies in the field. Since BERT [10], one of the most popular transformer architectures, and LM were launched at the end of 2018, we searched for studies from 2019 onward. The primary databases used for this review were Scopus [16] and the Web of Science Core Collection (WOSCC) [17] since both databases are renowned for indexing a wide array of peer-reviewed journals across multidisciplinary fields [18,19]. While acknowledging that additional databases might offer further insights, the significant overlap with these resources ensures that relevant studies are unlikely to be missed.

The criteria were defined to include studies focused on continuing the pretraining of LMs to achieve health care–domain LMs or studies focused on creating IE models within the health care field. Therefore, we formulated a query that includes the training or fine-tuning LMs or IE Models within the context of health care or similar, using EMRs or EHRs as data.

Since there is a significant semantic similarity between LMs and LLMs, we decided to exclude the second from the search query because it has a different purpose from the aim of our study. LLMs are typically composed of more than 7B parameters and are suited for text generation. LMs are models that are not, by themselves, suited to perform any downstream NLP task, needing to be readjusted or fine-tuned with labeled data to be able to perform downstream tasks.

Our final query is as follows: “(“Language Model” OR “Masked Language Model” OR “Information Extraction” OR “Content Extraction”) AND (“EHR” OR “EMR” OR “Electronic Health Record” OR “Electronic Medical Record”) AND (“Fine-Tuned” OR “Fine-tuning” OR “Training” OR “Trained”) AND (“Healthcare” OR “Health Care” OR “Clinical” OR “Medical”) AND NOT (“Large Language Model” OR “LLM”).”

According to our objectives, a study was considered valid if it documented a continuation of the pretraining of an LM to create a health care LM or if it focused on the creation of health care IE models.

### Study Selection

To minimize the risk of bias in the study selection, the process was conducted independently by 3 researchers. A total of 2 researchers were responsible for reading and judging the studies according to the inclusion criteria, while the third researcher was involved in cases of disagreement.

### Data Charting and Synthesis

A data-charting form was jointly developed by two reviewers to extract relevant information from the selected studies systematically. The form included variables such as study title, year of publication, language focus (English or non-English), domain adaptation techniques for Transformer-based models, healthcare-specific information extraction tasks, evaluation metrics used, and the specific health-related entities being extracted. Both reviewers independently charted the data to ensure comprehensive coverage of healthcare language models in English and non-English languages, with particular attention to languages other than English (referred to as non-English). Discrepancies in the extracted data were discussed and resolved through consensus. As the review progressed, the data-charting form was iteratively updated to capture emerging themes, especially regarding the disparity between language resources and technological development for healthcare information extraction across different languages.

## Results

**Figure 1:**
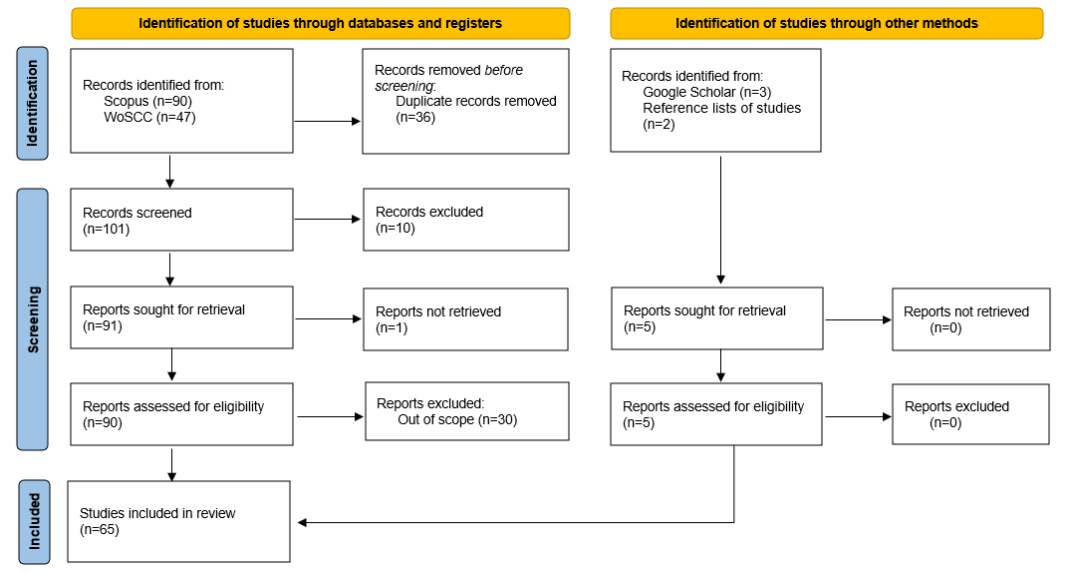
PRISMA-ScR (Preferred Reporting Items for Systematic reviews and Meta-Analyses extension for Scoping Reviews) workflow diagram. WOSCC: Web of Science Core Collection.

The query retrieved 137 papers, with the vast majority of these studies being retrieved from Scopus, adding up to 90 when compared with the 47 studies WOSCC has yielded. The PRISMA-ScR methodology was then followed, as seen in Figure 1. Since we included studies from sources beyond the 2 selected databases, we adhered to the updated PRISMA-ScR guideline [20]. In the following subsections, we explained the decision to include studies by other methods.

The first step was to identify and remove duplicated papers, resulting in 101 studies. Following a screening of titles and abstracts, 10 records were deemed out of scope, and 1 could not be retrieved, leaving us with 90 fully reviewed studies.

After screening all the papers that matched our criteria, we realized that 30 of them did not meet our inclusion criteria. Some studies referred to the fine-tuning of pretrained LMs for tasks unrelated to IE, or they lacked relevant information to contribute to this study, or even though we excluded them from our search query, they mentioned the use of LLMs.

As we were focused on the Portuguese language, our study also emphasized the geographical domain, with an aim to comprehend the medical data language used in health care LMs. Table 1 resumes the studies focused on the pretraining of LMs separated by the language of their data.

From the reading of Table 1, we can understand that English is the main language, which can be explained by the much higher availability of English data and the overwhelming presence and applicability of this language throughout the world. However, we notice that in the Chinese language, there are studies attempting to fill the gap of being non-English, creating in-domain LMs aware of their benefits. We also found studies in Brazilian Portuguese (PT-BR), Spanish, and PT-PT, and we acknowledge that there might be other studies in different languages, even though they did not match our search query criteria.

**Figure 2:**
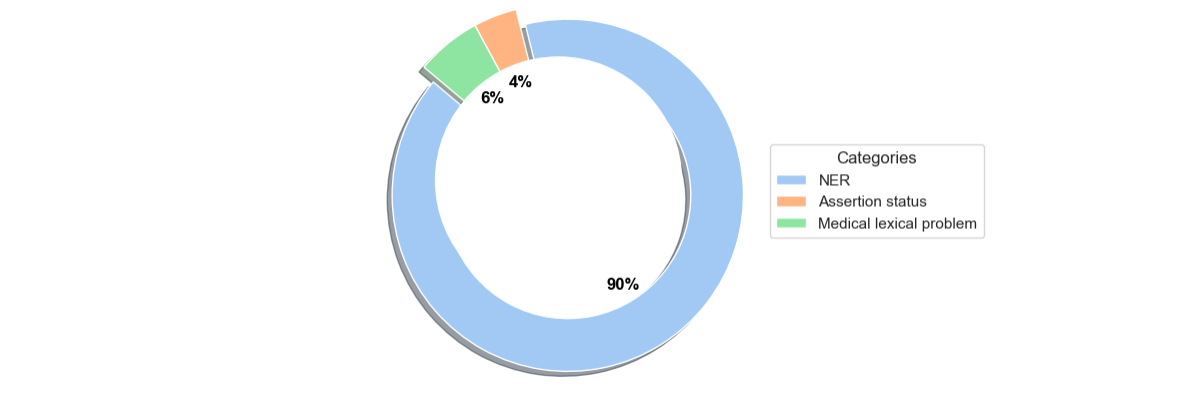
Distribution of health care information extraction (IE) studies by topic. NER: named entity recognition.

Changing the view for the health care IE studies, Figure 2 resumes the distribution of studies by topic.

From the reading of Figure 2, NER appears as the main topic on the IE, with only 2 studies performing Assertion Status and 3 studies focused on solving medical lexical problems.

To provide a more in-depth review of each study, we present the subsequent 3 subsections where we differentiate between non-Portuguese health care LMs, Portuguese health care LMs, and health care IE models.

**Table 1:** Studies for pretraining language models (LMs) were reviewed by their data language.

Medical data language	Reference	Studies, n
English	[11,12,21-25]	7
Chinese	[26-29]	4
Brazilian Portuguese	[30,31]	2
Spanish	[32]	1
European Portuguese	[33]	1

### Non-Portuguese Health Care Language Models

A study by Zhou et al [12] introduces CancerBERT, a domain-specific LM, that resulted from continuing the pretraining of the BlueBERT model [34] with a cancer corpus, resulting in various checkpoints of CancerBERT. The evaluation was performed for the cancer phenotyping NER task, with the results showing that the CancerBERT model pretrained with the cancer corpus outperformed the checkpoint using the original BERT [10] vocabulary.

A similar approach was conducted in a study by Mao et al [11], where the objective was to predict the risk of disease progression from Mild Cognitive Impairment to Alzheimer disease. A BERT model specifically tailored for Alzheimer disease (ie, AD-BERT) was pretrained with clinical notes, and its comparison with other models in experiments showed the benefits of domain adaptation.

Within the same scope, the identification of fall incidents from EHRs is discussed in the study by Fu et al [21]. A context-aware LM, BERT-based, was trained and integrated into a hybrid architecture along with post hoc heuristic rules. The performance of the BERT-based model was compared with DL methods, and the conclusions highlighted that the BERT model achieved superior results in identifying fall events.

In a study by Wang et al [26], a Chinese medical text corpus was used to pretrain BERT and obtain the Chinese BERT model. The results were aligned with previous studies, and domain adaptation demonstrated better results than traditional DL models and other pretrained LMs.

Studies by Roitero et al [22] and Agnikula Kshatriya et al [23] once again mention the pretraining of BERT models on a medical corpus, achieving comparable or better performance than state-of-the-art models. In a study by Zhang et al [24], an unsupervised adversarial domain adaptation framework with a pretrained LM for clinical event sequences is presented. Another example can be found in a study by Chen et al [25], where a contextual LM is used in combination with rule-based preprocessing methods to develop a model for *ICD-10* (*International Statistical Classification of Diseases, Tenth Revision*) multilabel classification. The results demonstrate superiority over state-of-the-art models. Studies by Wen G et al and Wen C et al [27,28] refer to the training of a domain-specific pretrained LM on unlabeled medical data, with the evaluation being made through NER.

In the process of reviewing all the papers, we observed references to papers that aligned with our requirements despite not being explicitly included within our search query criteria. It is the case of studies by Zhang et al [29] and Carrino et al [32]. Carrino et al [32] present a large-scale biomedical Spanish LM, where the models were pretrained from scratch, using a RoBERTa [35] base model, and then fine-tuned on 3 clinical NER tasks. The comparison between general-domain and other available Spanish clinical models revealed the superiority of the models presented in the paper. Zhang et al [29] share a similar scenario with BERT being pretrained on Chinese biomedical corpora, and MC-BERT, an in-domain LM, was developed. The results are consistent with previous studies, with MC-BERT outperforming BERT-based models in all evaluated tasks.

### Portuguese Health Care Language Models

Our search query did not retrieve any studies for the Portuguese language. To address this scarcity of studies and since it is one of the objectives of this research, we carried out a broader search on Google Scholar [36] to include studies that mentioned the creation of Portuguese health care LMs.

The PT-BR language has already presented several studies, with BioBERTpt [30] being one example. The authors used clinical notes and biomedical abstracts to pretrain 3 BERT-based checkpoints that were fine-tuned for the NER task to assess their performance. The results align with others, showing that the in-domain models achieved better performance. Another example is the study by Schneider et al [31], where several clinical BERT-based checkpoints were developed resulting from the continuation of the pretraining of BERTimbau [35], mBERT [11], and all 3 BioBERTpt checkpoints on 150,000 clinical narratives from cardiology ambulatory. The results of fine-tuning for NER align with previous studies, demonstrating that the in-domain models outperformed general LMs.

For the PT-PT language, we found the literature to be scarcer, with only 1 study being found that mentioned the continuation of the pretraining of an LM to achieve health care–domain LM. Coutinho and Martins [33] propose a BERT-based model for assigning *ICD-10* codes to causes of death by using BERTimbau and continuing its pretraining with death certificates. The evaluation was made through NER, with all the checkpoints involved being fine-tuned for the classification task, and the findings indicated that transformer models could produce promising outcomes for health care tasks involving the analysis of relatively short documents.

### Health Care IE Models

To better organize this section, we decided to categorize the studies by topic. Therefore, the first subsection presents NER studies where the authors attempted to automatically identify and extract medical information. The second subsection contains Assertion Status models, where entities are classified according to their status (present or absent), and finally, the third subsection presents studies that attempt to solve medical lexical problems.

### NER

Zhou et al [37] evaluated the performance of CancerBERT along with ML models for the breast cancer phenotype extraction task, with the results proving that CancerBERT has superior learning ability and generalizability for this task. Rahman et al [38] refer to the use of BERT to identify the presence of a diagnosis in EHRs. With BERT’s ability to understand the context of text and based on conditions presented in EHRs, a pipeline was successfully designed to identify EHRs with the presence of a diagnosis, reducing the manual note review load. Crema et al [39], use an Italian biomedical BERT model, fine-tuning it for the NER task with the entities of interest, including diagnoses, symptoms, drugs, and medical assessments, achieving an *F*_1_-score of nearly 0.85 values. Entity-BERT was introduced in the study by Lu et al [40], a DL-based model for entity IE that is capable of recognizing entities such as medical terminologies, disease names, or drug information. Zhang et al [41] propose the combination of data augmentation and domain information using the Adapter Transformer Encoder Model for Clinical Event Detection. It uses the BioBERT model to generate word-level features, addressing the issue of many obscure professional terms in EMRs leading to poor recognition performance of the model. The results were reported to be superior to those of other existing models. A multilingual transformer was fine-tuned in a study by Kim et al [42], where researchers successfully extracted alcohol-related information from unstructured clinical notes with an extraction accuracy of 0.84 as measured by the macro *F*_1_-score. Kormilitzin et al [43], initially pretrained a model on the task of predicting the next word and subsequently fine-tuned it for the NER task, extracting various categories of drugs and achieving performance with an *F*_1_-score above 0.95 values. Solarte-Pabón et al [44] evaluate the fine-tuning of several pretrained LMs for the NER task, aiming to identify breast cancer concepts in the Spanish language. The results show that BERT-based and RoBERTa-based LMs exhibit competitive performance on this task. Liu et al [45] propose the use of BERT-BiLSTM-CRF for the NER task of rheumatoid arthritis vocabulary and then MC-BERT for the entity extraction task, with results showing *F*_1_-scores above 90%. Wang et al [46] compare the use of 4 pretrained transformer-based LMs fine-tuned for the NER task with a baseline regular expression model in order to extract ophthalmic examination components, demonstrating that transformers achieve superior results. In the study by Singh et al [47], a pretrained transformer-based LM was fine-tuned with cardiac magnetic resonance imaging annotations to effectively extract measurements from clinical reports, and it achieved high extraction performance without requiring heuristics or expert annotations.

Several studies focus on extracting information about family history, such as studies by Kim et al [48], He et al [49], Silva et al [50], Dai et al [51], and Zhan et al [52]. They use ML methods, incorporating rule-based approaches, multitask-based artificial neural networks (ANN), attention-based neural networks, and even combinations such as convolutional neural networks (CNNs) BiLSTM and BERT. The goal was to automatically extract entities such as people’s names, residence, birth date, or death date, and in some cases, there is an additional subtask related to relation extraction, which involves identifying relationships between family members. Overall, the results have proven to be satisfactory, particularly in the NER task.

CNNs are highly popular methods in the scientific community for extracting clinical information and studies by Yang et al [53], Santus et al [54], Mahajan and Rana [55], and Landlosi et al [56] primarily used them, often supplemented with rule-based approaches or feature optimization in some cases. The use of these methods lies in extracting clinical information from EHRs, tasks that could be time-consuming if done manually. Within the broader category of neural networks, RNNs are also a method used for IE in which the authors of studies [57-66] all use RNNs, with BiLSTM-CRF (Bidirectional Long Short-Term Memory - Conditional Random Field) being a very popular network among these studies. The main topics extracted include terms related to specific diseases, drug names with associated attributes (dosage, frequency, duration, route, and condition), adverse drug events, the presence of a diagnosis, or even important information in medical image reports, with the results globally proven to be promising.

Studies [67-69] use ML methods, with the first focusing on automatically classifying the outcomes of specific tasks related to the clinical conditions of stroke survivors, the second aiming to extract useful information in abdominopelvic radiology reports, and the third one focused on extracting travel history mentions from clinical documents. In Malmasi et al [70], the use of different methods to extract low-prevalence concepts is discussed, specifically in the case of insulin rejection by patients with attempts at both sentence-level and token-level approaches using ML and DL methods, but the results showed that it is challenging to automatically identify low-prevalence concepts. Similar proposals have been presented in studies [71-79] using spaCy’s [80] pipeline for IE, contextual embeddings such as embeddings from language models (ELMo) [81] and BERT, position-attention mechanisms, knowledge graph embeddings, word segmentation models, or even NLP models developed using Java for extracting medical information, for example, extracting details related to drugs, drug attributes, or diagnoses.

In Lee and Uppal [82], a web-based summarization and visualization tool is introduced for extracting salient information from clinical and biomedical text, featuring sentence ranking by relevance and facilitating early medical risk detection in clinical settings. Chen et al [83] aimed to create a model to extract concept embeddings from EHRs for disease pattern retrieval and subsequent classification tasks.

### Assertion Status

Sykes et al [84] address the issue of negation and non-negation of clinical terms in EHRs. It is an Assertion Status case, in which the text can be characterized by cases where diseases are stated to be absent or only hypothesized. In this study, they propose various methods to address this issue, including rule-based, ML, or DL approaches, and all proposals yielded good results in a test set, achieving an *F*_1_-score of more than 0.95. In Chaturvedi et al [85], a corpus annotated with mentions of pain was developed, considering the presence or absence of pain. It is another example of an Assertion Status problem aimed at facilitating further studies using the corpus to better understand how pain is mentioned in clinical notes.

### Medical Lexical Problems

From a different perspective, there have been studies focusing on medical lexical problems. Newman-Griffis et al [86] discuss the presence of ambiguous words and attempts to normalize medical concepts to standardize vocabularies, while the study by Jaber et al [87] addresses the problem of the frequent use of abbreviations by proposing a method, by fine-tuning a pretrained LM, to successfully disambiguate clinical abbreviations. Lee et al [88] propose a typographical error correction model that considers context, based on a masked LM, to address the issue of typographical errors in real-world medical data. They conclude that typographical errors in unstructured text negatively impact the performance of NLP tasks, and their method is robust and applicable in real-world environments.

## Discussion

### Principal Findings

Continuing the pretraining of LMs to develop health care LMs has proven beneficial. The most common method to evaluate this approach is by fine-tuning both the baseline and the in-domain LM on downstream NLP tasks and comparing the results.

In IE models, NER is the most popular topic aimed at automatically identifying and extracting medically relevant information. Transformers are the preferred technology for this purpose, with fine-tuning of medical LMs consistently achieving superior results.

To conclude our SLR, we engaged in a deeper discussion divided into health care LMs and health care IE models.

### Health Care Language Models

On a global scale, we have identified numerous studies that continued the pretraining of LMs to develop domain-specific LM, specifically medical LMs. In general, the findings across almost all of these studies substantiate the advantages of in-domain training before undertaking any other downstream tasks. The favorite evaluation task is NER, with almost every study mentioning the fine-tuning of LMs for the NER task.

As shown in Table 1, English and Chinese are the languages with the most studies and published models due to the available resources in terms of data and hardware power. The level of domain adaptation for these languages is more advanced, with dedicated health care LMs developed for specific diseases such as Alzheimer Disease-BERT [11] and CancerBERT [12], which represent very focused domains. These studies offer advantages by achieving better performance in extracting specific concepts from textual data related to these diseases compared with general health care LMs.

For non-English languages, the process is not so developed, which can be considered as expected since they have their known limitations, such as the scarcity of data and resources available. Nevertheless, there have been concerted efforts to create general health care LMs, underscoring the community’s recognition of the use of these models. The Portuguese language fits this context, and despite initial strides that have already been taken, there exists ample room for improvement, particularly in the context of PT-PT where the only published study is [33], yet, to the best of our knowledge, the model is not publicly available.

Non-English languages, particularly Portuguese, should draw inspiration from advancements and results in medical domain adaptation studies. Despite limited resources and available data, efforts should first focus on creating general medical LMs. In a subsequent phase, efforts should be directed toward narrowing down to specific diseases while performing domain adaptation. This approach ensures that knowledge previously acquired by the LMs is refined within the medical domain and then adapted to smaller medical domains without losing the previously acquired knowledge completely. This initiative aims to foster the development of AI technologies in Portuguese, thereby promoting health care equality and access in languages with fewer resources. These models can be further fine-tuned for medical NLP tasks, such as IE, aimed at automatically identifying or highlighting specific information or structuring medical information extracted from textual data for ML analysis to aid health care professionals.

### Health Care IE Models

Several methods have been used to create health care IE models. The most common method is the use of transformers, followed by the application of other DL and ML methods (Table 2). As previously discussed, the most popular topic was NER, where authors attempted to identify and extract medically relevant information.

The results indicate that the most successful approach involves using pretrained LMs fine-tuned for IE tasks, benefiting from the contextual understanding of the text to achieve better results. The most commonly identified entities were diagnoses or diseases and drugs, along with specific phenotypes related to certain diseases.

**Table 2:** Number of studies used per method.

Methods	Articles
Transformers	16
Other DL^a^	15
RNN^b^	10
Other ML^c^	9
CNN^d^	5
Rule-based	4

^a^DL: deep learning.

^b^RNN: recurrent neural network.

^c^ML: machine learning.

^d^CNN: convolutional neural network.

It is also relevant to mention that in our query, 2 studies were focused on Assertion Status. This task involves classification at the sentence level aimed at assessing an entity based on its presence or absence in the text. Examples of absence include negation or hypothesizing medical information. From another perspective, to address the problems presented by medical text, we also found 2 studies that propose solutions to disambiguate the multitude of abbreviations present in medical text and 1 study that presents a typo correction model. Both solutions aimed to improve text quality and seek to correct issues in the text that are considered inevitable by health care professionals. These 5 studies could also be seen as an improvement to NER results. The ones focused on correcting the text could be viewed as a preprocessing step that would enhance the understanding of the medical text, while the Assertion Status studies could help ascertain whether an identified entity is present or absent in a patient’s condition. When compared with the distribution of NER, these 2 topics lack development, as together, they account for only 10% of the health care IE studies found. The community would benefit from more studies using different technologies and identifying new challenges to be solved.

### Conclusions

Our SLR highlights the benefits of in-domain training for health care LMs and the effectiveness of transformers in IE tasks, addressing a research gap regarding the lack of studies on health care LMs. Transformers excel in NER, identifying diagnoses, diseases, drugs, and phenotypes. English and Chinese lead in research and LM development, while non-English languages such as Portuguese show promise but need exploration. Challenges include Assertion Status and text disambiguation, necessitating diverse methodologies and research in health care IE.

We have identified several health care–domain LMs, but there is a clear gap for non-English languages where the data and resources available are low. There is much to improve in those languages, with Portuguese being an example. The benefits of creating a medical-domain LM are already proven, and the health care sector could benefit greatly from a symbiosis with AI. Therefore, non-English languages should be motivated by the scarce studies already published and try to replicate them for their own language in order to fill this existing gap.

From another point of view, the use of transformers appears to be the better technique to automatically identify medical information. Despite the annotation process for any supervised learning task being very time-consuming, transformers achieve better results on fewer annotations, making their usage on new tasks relatively easier. This task also benefits from an in-domain medical LM. The entities most commonly extracted are diagnosis or disease, posology-related entities, symptoms, and phenotypes related to specific diseases.

Despite our belief that this was the right choice, we highlight the 2 databases that we searched, and we acknowledge that, despite our best efforts, there is always a possibility that not all relevant papers will be found when formulating a query. These are the limitations of our study. The chosen timeframe may also limit the availability of relevant studies, even though we believe it is the right timeframe to include studies that establish the current state-of-the-art with new technologies. While we focused on the Portuguese language, we acknowledge that our conclusions cannot be generalized to all non-English languages. However, other languages with similar characteristics in terms of available data and resources can certainly gain insights from this SLR.

Globally, the development and research in these topics for the English language are very advanced compared with non-English languages. In English, several studies have been presented that perform domain adaptation for smaller domains, such as specific-disease LMs, which have improved results in extracting medical information related to these diseases. The next steps should involve continuing the pretraining for different medical areas or diseases to ensure the most comprehensive coverage with LMs. In addition, fine-tuning the already available models to meet the specific requirements of health care professionals is essential.

Non-English languages are still performing domain adaptation for general domains, such as medical or biomedical fields, and should be motivated by these studies to overcome the barriers inherent in their respective language. In the next step, they should focus on performing domain adaptation, aiming to narrow down to specific medical areas or diseases. They should strive to replicate studies on Assertion Status or even those focused on resolving the frequent presence of abbreviations and typos in the text. In non-English languages where there is a scarcity of available data, it would be beneficial to have open corpora, even if distributed under licenses that protect data privacy, to enable more researchers to develop models.

These types of studies are important to assess and guide the development of non-English languages trying to bridge the gaps and capitalize on the opportunities provided by these technologies to promote equity and improve access to health care all over the world. The differences in the available data and resources are almost impossible to correct but at least should be minimized.

This effort aims to harness AI to enhance health care by developing advanced LMs tailored for non-English languages, thereby supporting health care professionals with decision-making tools that alleviate their workload and improve patient care indirectly.

## Appendix

Multimedia Appendix 1 PRISMA-ScR checklist.

## Abbreviations

AI: artificial intelligence
ANN: artificial neural network
BERT: Bidirectional Encoder Representations from Transformers
BiLSTM-CRF: Bidirectional Long Short-Term Memory - Conditional Random Field
CNN: convolutional neural network
DL: deep learning
EHR: electronic health record
EMR: electronic medical record
ELMo: embeddings from language models
ICD-10: International Statistical Classification of Diseases, Tenth Revision
IE: information extraction
LLM: large language model
LM: language model
ML: machine learning
NER: named entity recognition
NLP: natural language processing
PRISMA-ScR: Preferred Reporting Items for Systematic reviews and Meta-Analyses extension for Scoping Reviews
PT-BR: Brazilian Portuguese
PT-PT: European Portuguese
RNN: recurrent neural network
SLR: systematic literature review
WOSCC: Web of Science Core Collection
